# Grey and White Matter Volume Changes after Preterm Birth: A Meta-Analytic Approach

**DOI:** 10.3390/jpm11090868

**Published:** 2021-08-30

**Authors:** Benita Schmitz-Koep, Bernhard Haller, Pierrick Coupé, Aurore Menegaux, Christian Gaser, Claus Zimmer, Dieter Wolke, Peter Bartmann, Christian Sorg, Dennis M. Hedderich

**Affiliations:** 1 Department of Neuroradiology, School of Medicine, Technical University of Munich, Ismaninger Str. 22, 81675 Munich, Germany; aurore.menegaux@tum.de (A.M.); claus.zimmer@tum.de (C.Z.); christian.sorg@tum.de (C.S.); dennis.hedderich@tum.de (D.M.H.); 2 TUM-NIC Neuroimaging Center, School of Medicine, Technical University of Munich, Ismaninger Str. 22, 81675 Munich, Germany; 3 Institute of Medical Informatics, Statistics and Epidemiology, School of Medicine, Technical University of Munich, Ismaninger Str. 22, 81675 Munich, Germany; bernhard.haller@tum.de; 4 Laboratoire Bordelais de Recherche en Informatique (LaBRI) UMR 5800, CNRS, Bordeaux INP, University of Bordeaux, F-33400 Talence, France; pierrick.coupe@u-bordeaux.fr; 5 Department of Psychiatry, University Hospital Jena, Am Klinikum 1, 07747 Jena, Germany; christian.gaser@uni-jena.de; 6 Department of Neurology, University Hospital Jena, Am Klinikum 1, 07747 Jena, Germany; 7 Department of Psychology, University of Warwick, University Road, Coventry CV4 7AL, UK; D.Wolke@warwick.ac.uk; 8 Warwick Medical School, University of Warwick, University Road, Coventry CV4 7AL, UK; 9 Department of Neonatology, University Hospital Bonn, Venusberg-Campus 1, 53127 Bonn, Germany; Peter.Bartmann@ukbonn.de; 10 Department of Psychiatry, School of Medicine, Technical University of Munich, Ismaninger Str. 22, 81675 Munich, Germany

**Keywords:** brain development, grey matter volume, white matter volume, structural magnetic resonance imaging, preterm birth

## Abstract

Cross-sectional studies have reported lower brain grey matter volumes (GMV) and white matter volumes (WMV) in preterm (PT) born individuals. While large MRI studies in the normative population have led to a better understanding of brain growth trajectories across the lifespan, such results remain elusive for PT born individuals since large, aggregated datasets of PT born individuals do not exist. To close this gap, we investigated GMV and WMV in PT born individuals as reported in the literature and contrasted it against individual volumetric data and trajectories from the general population. Systematic database search of PubMed and Web of Science in March 2021, and extraction of outcome measures were conducted by two independent reviewers. Individual data on full-term (FT) controls was extracted from freely available databases. Mean GMV, WMV, total intracranial volume (TIV), and mean age at scan were the main outcome measures. Of 532 identified records, nine studies were included with 538 PT born subjects between 1.1 and 28.5 years of age. Reference data was generated from 880 FT controls between 1 and 30 years of age. GMV was consistently lower in PT born individuals from infancy to early adulthood with no evidence for catch-up growth. While GMV changes followed a similar trajectory as FT controls, WMV was particularly low in adolescence after PT birth. Results demonstrate altered brain volumes after PT birth across the first half of lifespan. Future studies should address this issue in large aggregated datasets of PT born individuals.

## 1. Introduction

Preterm (PT) birth, defined as birth <37 weeks of gestation, has a high worldwide prevalence of almost 11% [1]. Prematurity is related to alterations in brain development in general, and in grey matter volume (GMV) and white matter volume (WMV) in particular, which has been documented at various age groups from infancy and childhood until early adulthood [2,3,4,5,6,7].

Large population studies and open datasets have led to a better understanding of brain development and aging in the general population across the lifespan [8,9,10]: GMV rapidly increases during infancy and early childhood until it peaks at school age. A fast decrease until around 40 years of age follows with a subsequent plateau phase. Lastly, there is an accelerated decrease of GMV around 80 years of age. Development of WMV follows an inverted U-shape. After fast growth in early ages, WMV peaks at around 30 to 40 years of age followed by a volume decrease. Characterization of age-dependent grey and white matter development in healthy subjects has enabled modelling pathological alterations in patients. For example, deviations from normal brain volume trajectories have been described in pathologic states such as Alzheimer’s disease and may be used as a diagnostic tool [11].

However, brain structure alterations after PT birth have mostly been studied in cross-sectional designs with narrow age ranges. Data from longitudinal studies with several magnetic resonance imaging (MRI) examinations are scarce and shared large datasets do not exist for PT populations. Hence, aggregated evidence about brain growth trajectories of the lifespan after PT birth remains elusive.

To close this gap, we investigated GMV and WMV changes observed in cross-sectional studies of PT subjects over the first half of lifespan from infancy to early adulthood and contrasted it against brain growth trajectories of a large normative cohort of FT individuals.

## 2. Materials and Methods

This meta-analysis was conducted following the Preferred Reporting Items for Systematic Reviews and Meta-Analyses (PRISMA) guidelines, see Table S1 [12]. It was not registered. A review protocol was not prepared.

### 2.1. Search Strategy

The electronic databases PubMed and Web of Science were systematically searched for articles published before 30 March 2021. Key words: (birth OR born) AND (preterm OR prematur *) AND (magnetic resonance imaging OR MRI) AND (brain OR intracranial) AND (volume).

### 2.2. Study Selection Criteria

The following inclusion criteria were used to assess eligibility: (1) participants were born PT (<37 weeks of gestation); (2) MRI was used to determine mean total intracranial volume (TIV), total GMV, and total WMV; (3) mean age at MRI scan was reported and >1 year; and (4) the study was published in a peer-reviewed journal. If multiple studies reported on volumetric data of the same cohort at the same age, the study with the larger sample was included. Records were screened by two independent reviewers.

Studies with mean age lower than one year were excluded because of particular rapid brain growth in this period, in which small changes in postnatal age correspond to large volumetric changes [13].

### 2.3. Data Collection Process and Data Extraction

Data was extracted from the studies by two independent reviewers using a standardized form including author and year of publication, sample size, age at MRI scan (in years), mean and SD of TIV, GMV and WMV (in cm^3^), gestational age (GA, in weeks), birth weight (BW, in grams), percentages of male participants, year of birth, country of origin, and methodology of brain volume estimation. If PT samples were separated into groups (e.g., GA subgroups, positive or normal cranial ultrasound (cUS) findings and with or without intrauterine growth restriction (IUGR), volumes were reported and analyzed separately. If age was reported in months, it was divided by twelve to obtain age in years. If volume was reported in mm^3^, it was divided by 1000 to obtain volume in cm^3^. If volume was reported in dm^3^, it was multiplied with 1000 to obtain volume in cm^3^. One study [7] reported mean GMV/TIV-ratio and mean WMV/TIV ratio. Mean GMV and WMV values were calculated from these ratios. If BW was reported in kilograms, it was multiplied with 1000 to obtain weight in grams.

### 2.4. Data on Full-Term Controls

Data on FT controls was extracted from freely available databases: Cincinnati MR Imaging of NeuroDevelopment (C-MIND, https://research.cchmc.org/c-mind/ (accessed on February 2015)), National Database for Autism Research—NIH MRI Study of Normal Brain Development, Pediatric MRI Data Repository (NDAR-NIHPD, https://www.bic.mni.mcgill.ca/nihpd_info/info2/data_access.html (accessed on February 2015)), International Consortium for Brain Mapping (ICBM, http://www.loni.usc.edu/ICBM/ (accessed on February 2015)), and Information eXtraction from Images (IXI, http://brain-development.org/ixi-dataset/ (accessed on February 2015)). TIV, GMV and WMV were calculated from these four datasets as previously described by Coupé et al. [10] Overall, 880 FT controls between 0.7 and 30 years of age were included: 236 participants from C-MIND (mean age = 8.4 years, age range = 0.7–18.9 years, male = 45.3%), 375 from NDAR-NIHPD (mean age = 11.9 years, age range = 1.1–29.0 years, male = 53.6%), 169 from ICBM (mean age = 23.9 years, age range = 18–30 years, male = 52.7%), and 100 from IXI (mean age = 25.7 years, age range = 20.0–29.8 years, male = 44%).

### 2.5. Statistical Analysis

Statistical analysis was performed with R [14]. A nonlinear regression model (Model 6, the cubic hybrid model, from Coupé et al. [10]: $\mathrm{Vol}\;=\;\beta_{4}\;\left(1-e^{-{\mathrm{Age}/\beta }_{5}}\right)+\beta_{0}+\beta_{1}\mathrm{Age}+\beta_{2}{\mathrm{Age}}^{2}+\beta_{3}{\mathrm{Age}}^{3}+\varepsilon$) was used to model GMV, WMV, TIV, GMW/TIV, and WMV/TIV for FT controls in dependence of age using the R library minpack.lm [15]. Grey areas surrounding the curves show 95%-confidence intervals (CI), dotted lines show 95%-prediction intervals (PI), which were calculated using the R library propagate [16].

## 3. Results

### 3.1. Study Selection and Characteristics

**Figure 1:**
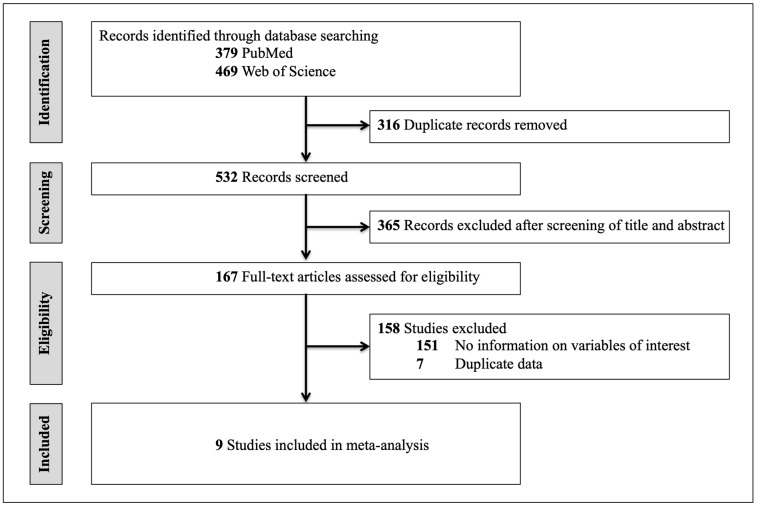
Flowchart of study selection.

The search strategy identified 532 records (Figure 1, Table 1). After screening of abstract and title, 167 articles were identified as potentially relevant. Based on the inclusion criteria, nine studies were eligible for the meta-analysis [5,7,17,18,19,20,21,22,23]. Most of the studies excluded did not report on all variables of interest, i.e., TIV, GMV and WMV. For example, a recent study tracking regional brain growth up to age 13 in children born term and very preterm (VP) appeared to meet the inclusion criteria. However, while it did report mean age at scan, mean TIV and mean WMV, it did not report mean global GMV [24]. Therefore, it was excluded. Another study investigating regional brain volume abnormalities and long-term cognitive outcome in PT infants reported adjusted marginal mean of specific brain regions, however, it did not report mean TIV, WMV or GMV [25]. Therefore, it was excluded. Seven studies were excluded because they reported on data from cohorts that were already covered by other studies. In summary, 538 PT subjects collected in studies with mean age between 1.1 and 28.5 years, and data from 880 FT controls between 0.7 and 30 years of age were analyzed. Please see Figure 1 for a flowchart depicting study selection. Study characteristics of the nine studies included are shown in Table 1.

### 3.2. Development of Grey Matter Volume after Preterm Birth

**Figure 2:**
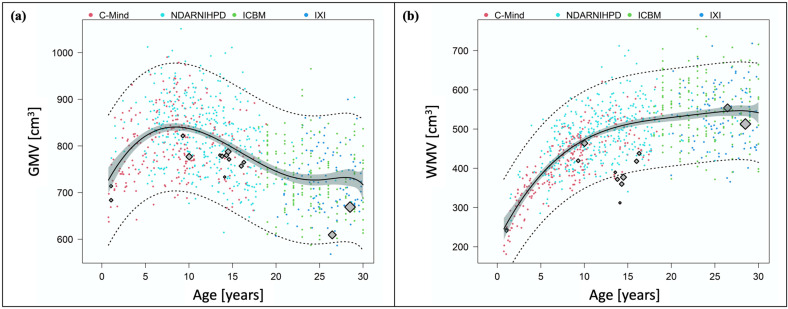
Mean absolute grey and white matter volumes after preterm birth compared to full-term controls. The four different datasets on FT controls are illustrated in different colors. C-MIND is presented in red, NDAR-NIHPD in in cyan, ICBM in green, and IXI in blue. Mean absolute volumes from the PT studies are included as diamonds. The size of each diamond is proportional to the sample size of the study. (**a**) Mean absolute GMV after PT birth compared to FT controls. The graph shows mean absolute GMV extracted from the PT studies contrasted against the GMV trajectory of FT controls with 95%-CI (grey areas) and 95%-PI (dotted lines). (**b**) Mean absolute WMV after PT birth compared to FT controls. The graph shows mean absolute WMV extracted from the PT studies contrasted against the WMV trajectory of FT controls with 95%-CI (grey areas) and 95%-PI (dotted lines).

Figure 2a illustrates mean absolute GMV extracted from the PT studies contrasted against individual GMV data of FT controls. Mean absolute GMV in all of the PT studies was below mean GMV in FT development. One study [20] reported mean absolute GMV of PT infants appropriate for gestational age (AGA) at about 1 year of age just within the 95%-CI. All other studies reported mean absolute GMV below the 95%-CI. The highest mean absolute GMV was reported at school age. While mean absolute GMV decreased between adolescence and early adulthood in both PT subjects and FT controls, this decrease appeared steeper in the PT studies. 

**Figure 3:**
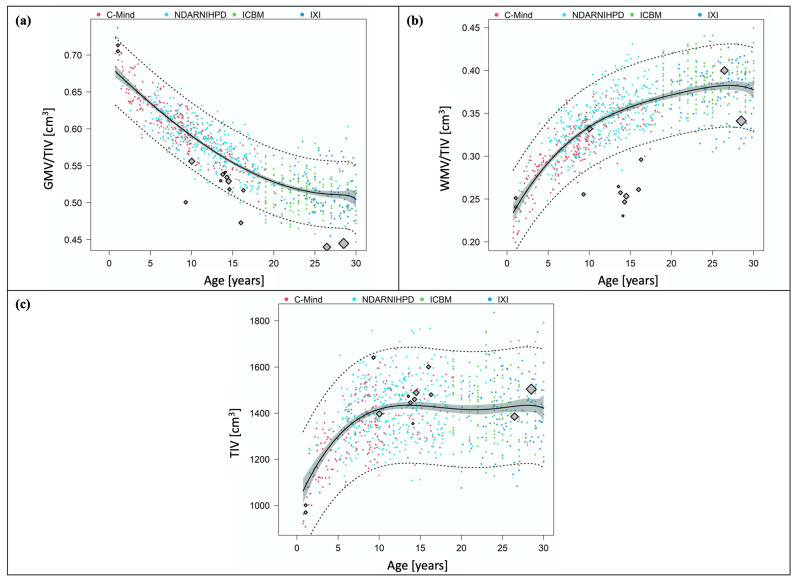
Relative grey and white matter volumes, and total intracranial volume after preterm birth compared to full-term controls. The four different datasets on FT controls are illustrated in different colors. C-MIND is presented in red, NDAR-NIHPD in in cyan, ICBM in green, and IXI in blue. Data points from the PT studies are included as diamonds. The size of each diamond is proportional to the sample size of the study. (**a**) Relative GMV after PT birth compared to FT controls. The graph shows GMV/TIV extracted from the PT studies contrasted against the GMV/TIV trajectory of FT controls with 95%-CI (grey areas) and 95%-PI (dotted lines). (**b**) Relative WMV after PT birth compared to FT controls. The graph shows WMV/TIV extracted from the PT studies contrasted against the WMV/TIV trajectory of FT controls with 95%-CI (grey areas) and 95%-PI (dotted lines). (**c**) TIV after PT birth compared to FT controls. The graph shows mean TIV extracted from the PT studies contrasted against the TIV trajectory of FT controls with 95%-CI (grey areas) and 95%-PI (dotted lines).

Figure 3a illustrates GMV/TIV, i.e., relative GMV, from the PT studies contrasted against individual data of FT controls. Relative GMV decreased from infancy through early adulthood. Relative GMV calculated from data on infants with and without IUGR reported by Padilla et al. [20] was above the curve describing FT development. All other values were below the 95%-CI. Data from three PT studies of school-aged children [5], adolescents with brain injury [19] and adults [17] were below the 95%-PI.

In summary, GMV was lower after PT birth from infancy through early adulthood while following a similar trajectory as FT controls.

### 3.3. Development of White Matter Volume after Preterm Birth

Figure 2b illustrates mean absolute WMV extracted from the PT studies contrasted against individual WMV data of FT controls. Except for one study in early adulthood [7], mean absolute WMV in all of the PT studies was below the curve describing mean FT development. Mean absolute WMV of infants with and without IUGR reported by Padilla et al. [20] was within the 95%-CI. All other studies reported mean absolute WMV below the 95%-CI. Mean absolute WMV reported in adolescence was particularly low, moving away from the curve describing mean FT development. 

Figure 3b illustrates WMV/TIV, i.e., relative WMV, from the PT studies contrasted against individual data of FT controls. Relative WMV calculated from data on infants with and without IUGR reported by Padilla et al. [20] and from data in early adulthood [7] was above the curve describing FT development. Data from one PT study at school age [18] was below the curve describing FT development but just within the 95%-CI. All other values were below the curve. The other PT study at school age [5] and all PT studies in adolescents were below the 95%-PI.

In summary, we observed particularly low WMV in adolescence, while WMV in infancy and early adulthood were closer to the trajectory describing FT development.

### 3.4. Total Intracranial Volume after Preterm Birth

Figure 3c illustrates mean TIV extracted from the PT studies contrasted against individual TIV data of FT controls. While TIV reported by some studies in infancy, childhood and early adulthood was below the curve describing FT development, other PT studies reported higher values.

## 4. Discussion

This meta-analysis investigated changes of GMV and WMV after PT birth compared to FT controls across the first half of lifespan. We found lower GMV after PT birth from infancy until adulthood, indicating lastingly altered brain development. GMV changes of PT born individuals followed a similar trajectory as FT controls with no evidence for catch-up growth. WMV was particularly low in adolescence after PT birth. Furthermore, the present study highlights the need for large longitudinal datasets to compare PT and FT brain development across the lifespan.

### 4.1. Changes of Grey and White Matter Volume after Preterm Birth across the First Half of Lifespan

We found lower GMV after PT birth compared to FT controls from infancy through early adulthood. Similar to their FT peers, GMV in PT subjects peaked at school age and decreased between adolescence and early adulthood. However, relative GMV in infancy was higher compared to FT peers, possibly reflecting delayed maturation. Yet this speculation is based on only one study. At school age, in adolescence and in early adulthood GMV was lower. There was no evidence for catch-up growth. Furthermore, WMV after PT birth was particularly low in adolescence.

Comparing our results to the scarce longitudinal data available is difficult because of methodological differences. Ment et al. [26] observed reduction in cerebral GMV and an increase in WMV between 8 and 12 years of age. However, compared to FT controls, there was lower GMV reduction and less WMV gain over time [26]. In contrast, our results show that GMV in PT individuals mostly follow the FT reference curve, albeit on an overall lower level and that WMV is particularly low in early adolescence. Mostly in line with our results, Parker et al. [27] reported significantly smaller GMV and WMV in VP subjects compared to FT controls in adolescence (15 years) and in early adulthood (19 years). Furthermore, they reported similar growth patterns between these two timepoints [27]. Karolis et al. [28] observed GMV alterations in VP subjects between adolescence (15 years), early adulthood (20 years) and adulthood (30 years), indicating accelerated brain maturation. Similarly, our findings of lower relative GMV at school age, in adolescence and early adulthood might indicate early aging. Consistent with our findings, Karolis et al. [28] found no evidence for developmental catch-up. In line with our results, de Kieviet et al. [29] found reductions in GMV and WMV compared to FT controls in their investigation of brain volume throughout childhood and adolescence in subjects born VP and with very low birthweight in a meta-analysis. 

Proposed reasons for aberrant brain development after PT birth include several processes: Inflammatory, hypoxic-ischemic and/or stress-related events are potential causes for injury to preoligodendrocytes, axons, thalamus, subplate neurons or migrating gamma-aminobutyric acid-ergic neurons [6,30]. Remarkably, our results suggest a significant impact of prematurity on WMV in adolescence. On a cellular level, pre-oligodendrocytes, precursors of mature oligodendrocytes, are critical for white matter myelination. Pre-oligodendrocytes are specifically vulnerable to insults, such as ischemia and inflammation, resulting in cell injury or death and subsequent replenishment but dysmaturation [6,30,31,32]. Hence, our results showing preferential alteration of WMV might emphasize the significance of pre-oligodendrocyte vulnerability and dysmaturation in the context of prematurity.

GMV and WMV changes have been associated with functional outcome such as cognitive development in children, adolescents, and young adults [5,7,25,33]. Therefore, characterization of GMV and WMV changes across the first half of lifespan could help identify PT individuals at increased risk for impaired cognitive development.

In conclusion, PT birth has lasting effects on the development of GMV and WMV compared to FT controls with particularly low WMV in adolescence. We found no evidence for GMV catch-up growth.

### 4.2. What Is Needed in the Future?

PT cohorts have been followed from birth and well investigated, however, to date longitudinal imaging data on PT subjects are scarce and only cover specific age ranges. Shared large datasets do not exist. Methodological differences in image processing as well as statistical analysis make it difficult to compare results. Thanks to large, open datasets on healthy subjects, FT developmental trajectories have been well characterized [10], facilitating insights in diseases affecting brain structure, for example Alzheimer’s Disease [11]. Similarly, PT developmental trajectories could be used to identify PT adults with accelerated aging at risk for cognitive decline and/or neurodegenerative diseases. To achieve these goals, access to original imaging data of large PT populations across all age groups as well as shared individual patient data with information on parameters like birthweight, duration of neonatal intensive care unit exposure, and days of mechanical ventilation, and information about possible brain damage, such as intraventricular hemorrhage, is critical.

### 4.3. Limitations

There are limitations of this meta-analysis. First, only few studies fit the inclusion criteria (see also Section 2.2 and Section 2.3). One reason for exclusion was that raw volumes were not reported. For example, one study [2] investigated GMV and WMV in adolescents born VP, however, not raw volumes but volumes after controlling for whole brain volume were reported. Furthermore, not TIV but whole brain volume was reported [2]. Another study [26] investigated longitudinal brain volume changes in PT and FT subjects during late childhood and adolescence, however, volumes were not reported as raw values but as model-based least square means. Furthermore, not TIV but whole brain volume was reported [26]. A third study [34] investigated brain volumes and developmental outcome in childhood following fetal growth restriction leading to VP birth, however, mean age at scan was not reported. Another reason for exclusion was the use of other analysis methods, for example, investigating regional group differences instead of global volumes [25].

Second, only data on limited age groups was available. Hence, PT volume changes of GMV and WMV in comparison to FT trajectories remain less clear in some age groups, for example between about 2 and 7 years. However, it is inherently difficult to obtain a brain MRI in this age group since this is generally not possible without general anesthesia.

Third, as mentioned above, different methods of processing images make it very difficult to compare data. All PT studies included in this meta-analysis used toolboxes within SPM (https://www.fil.ion.ucl.ac.uk/spm/) for segmentation, while FT images were processed with volBrain [35]. Volumes obtained with volBrain showed high correlation with volumes obtained by manual segmentations [35]. Comparing subcortical segmentation of volBrain with state-of-the-art methods like Freesurfer or the FSL-based tool FIRST, and with manual segmentation showed competitive results in terms of accuracy and reproducibility. However, the possibility of systematic error cannot be ruled out. Only access to raw imaging data could improve this major limitation and, as discussed, is needed. However, contrasting individual MRI scans and derived measures of GMV and WMV to reference data from open datasets is increasingly used in clinical routine.

Lastly, GMV and WMV are global measures representing brain development. However, determination of developmental changes of other measures such as gyrification and individual volumes of subcortical structures are necessary to characterize PT brain development, but data is not available.

## 5. Conclusions

In conclusion, GMV is lower after PT birth from infancy through early adulthood, indicating lastingly altered brain development. GMV changes were similar to the trajectory of FT controls with no evidence for catch-up growth. WMV was particularly low in adolescence after PT birth. Large longitudinal datasets are crucial to compare PT with FT brain development across the lifespan.

## Figures and Tables

**Table 1:** Characteristics of preterm studies included.

Author (Year)	Group	Sample (*n*)	Age at Scan (Years)	TIV Mean (cm^3^)	TIV SD (cm^3^)	GMV Mean (cm^3^)	GMV SD (cm^3^)	WMV Mean (cm^3^)	WMV SD (cm^3^)	GA (Weeks)	BW (g)	Male (%)	Year of Birth	Country of Origin	Methodology of Brain Volume Estimation
Pascoe (2019) [17]		150	28.5	1504	140	669	61	513	61	28.8	1077	41.3	1986	New Zealand	CAT12 toolbox (SPM12)
Lemola (2017) [18]		57	10.0	1397	148	777	70	464	67	29.7	1447	65.3	1998–2006	Switzerland	New segment toolbox (SPM8)
Meng (2016) [7]		85	26.5	1385		609.4		554		30.67	1356	55.3	1985–1986	Germany	VBM8 toolbox (SPM8)
Northam (2011) [19]	positive cUS	27	16	1601	222	757	57	418	46	27.1	1081	44.4	1989–1994	United Kingdom	VBM5 toolbox (SPM5)
Northam (2011) [19]	normal cUS	22	16.3	1480	193	765	65	438	49	28.1	1098	31.8	1989–1994	United Kingdom	VBM5 toolbox (SPM5)
Padilla (2011) [20]	IUGR	18	1.1 *	969.6	101.8	683.7	64.5	243.5	36.9	32.1	1060	38.9	2006–2007	Spain	VBM5 toolbox (SPM5)
Padilla (2011) [20]	AGA	15	1.1 *	1001.1	95.4	714.0	57.0	240.7	38.0	31	1580	73.3	2006–2007	Spain	VBM5 toolbox (SPM5)
Soria-Pastor (2009) [5]		20	9.3	1641.2	172.6	821.7	84.9	419.2	53.8	32.5	1754	55	1996–1998	Spain	SPM5
Narberhaus (2007) [21]	GA ≤ 27	9	14.1	1354.8	174.1	733.4	54.7	312.1	57.9	26.4	899	77.8	1983–1994	Spain	SPM2
Narberhaus (2007) [21]	GA 28–30	19	14.6	1488.5	164.9	771.3	133.2	377.2	57.9	29	1140	42.1	1983–1994	Spain	SPM2
Narberhaus (2007) [21]	GA 31–33	25	13.8	1445.3	146.4	778.1	72.1	372.0	52.1	31.7	1534	44	1983–1994	Spain	SPM2
Narberhaus (2007) [21]	GA 34–36	11	13.55	1473.3	148.4	780.3	69.9	389.8	45.1	34.6	2446	63.6	1983–1994	Spain	SPM2
Gimenez (2006b) [23]		50	14.5	1488.8	148.8	787.8	80.8	377.2	47.4	29.9	1327	48	1982–1994	Spain	SPM2
Gimenez (2006a) [22]		30	14.3	1460	140	780	70	360	50	29.1	1108	n.a.	n.a.	Spain	SPM2

Abbreviations: AGA, appropriate for gestational age; BW, birth weight; cUS, cranial ultrasound; GA, gestational age; GMV, grey matter volume; IUGR, intrauterine growth restriction; SD, standard deviation; TIV, total intracranial volume; WMV, white matter volume. * corrected age.

## Data Availability

Please see references for the studies included in Section 3.1, and for data on full-term controls in Section 2.4.

## Supplementary Materials

The following are available online at https://www.mdpi.com/article/10.3390/jpm11090868/s1, Table S1: PRISMA Checklist.

## References

[1] Chawanpaiboon S., Vogel J.P., Moller A.-B., Lumbiganon P., Petzold M., Hogan D., Landoulsi S., Jampathong N., Kongwattanakul K., Laopaiboon M. (2019). Global, regional, and national estimates of levels of preterm birth in 2014: A systematic review and modelling analysis. Lancet Glob. Health.

[2] Nosarti C., Al-Asady M.H.S., Frangou S., Stewart A.L., Rifkin L., Murray R.M. (2002). Adolescents who were born very preterm have decreased brain volumes. Brain.

[3] Nosarti C., Nam K.-W., Walshe M., Murray R.M., Cuddy M., Rifkin L., Allin M.P.G. (2014). Preterm birth and structural brain alterations in early adulthood. NeuroImage Clin..

[4] Inder T.E., Warfield S.K., Wang H., Hüppi P.S., Volpe J.J. (2005). Abnormal cerebral structure is present at term in premature infants. Pediatrics.

[5] Soria-Pastor S., Padilla N., Zubiaurre-Elorza L., Ibarretxe-Bilbao N., Botet F., Costas-Moragas C., Falcon C., Bargallo N., Mercader J.M., Junque C. (2009). Decreased regional brain volume and cognitive impairment in preterm children at low risk. Pediatrics.

[6] Volpe J.J. (2009). Brain injury in premature infants: A complex amalgam of destructive and developmental disturbances. Lancet Neurol..

[7] Meng C., Bäuml J.G., Daamen M., Jaekel J., Neitzel J., Scheef L., Busch B., Baumann N., Boecker H., Zimmer C. (2016). Extensive and interrelated subcortical white and gray matter alterations in preterm-born adults. Brain Struct. Funct..

[8] Hedman A.M., van Haren N.E.M., Schnack H.G., Kahn R.S., Hulshoff Pol H.E. (2012). Human brain changes across the life span: A review of 56 longitudinal magnetic resonance imaging studies. Hum. Brain Mapp..

[9] Giedd J.N., Blumenthal J., Jeffries N.O., Castellanos F.X., Liu H., Zijdenbos A., Paus T., Evans A.C., Rapoport J.L. (1999). Brain development during childhood and adolescence: A longitudinal MRI study. Nat. Neurosci..

[10] Coupé P., Catheline G., Lanuza E., Manjón J.V. (2017). Alzheimer’s Disease Neuroimaging Initiative Towards a unified analysis of brain maturation and aging across the entire lifespan: A MRI analysis. Hum. Brain Mapp..

[11] Coupé P., Manjón J.V., Lanuza E., Catheline G. (2019). Lifespan changes of the human brain in Alzheimer’s Disease. Sci. Rep..

[12] Moher D., Liberati A., Tetzlaff J., Altman D.G., Altman D., Antes G., Atkins D., Barbour V., Barrowman N., Berlin J.A. (2009). Preferred reporting items for systematic reviews and meta-analyses: The PRISMA statement. PLoS Med..

[13] Dobbing J., Sands J. (1973). Quantitative growth and development of human brain. Arch. Dis. Child..

[14] (2020). R: A Language and Environment for Statistical Computing.

[15] Elzhov T.V., Mullen K.M., Spiess A.-N., Bolker B. (2016). minpack.lm: R Interface to the Levenberg-Marquardt Nonlinear Least-Squares Algorithm Found in MINPACK, Plus Support for Bounds. https://CRAN.R-project.org/package=minpack.lm.

[16] Spiess A.-N. (2018). Propagate: Propagation of Uncertainty. https://cran.r-project.org/web/packages/propagate/propagate.pdf.

[17] Pascoe M.J., Melzer T.R., Horwood L.J., Woodward L.J., Darlow B.A. (2019). Altered grey matter volume, perfusion and white matter integrity in very low birthweight adults. NeuroImage Clin..

[18] Lemola S., Oser N., Urfer-Maurer N., Brand S., Holsboer-Trachsler E., Bechtel N., Grob A., Weber P., Datta A.N. (2017). Effects of gestational age on brain volume and cognitive functions in generally healthy very preterm born children during school-age: A voxel-based morphometry study. PLoS ONE.

[19] Northam G.B., Liégeois F., Chong W.K., Wyatt J.S., Baldeweg T. (2011). Total brain white matter is a major determinant of IQ in adolescents born preterm. Ann. Neurol..

[20] Padilla N., Falcón C., Sanz-Cortés M., Figueras F., Bargallo N., Crispi F., Eixarch E., Arranz A., Botet F., Gratacós E. (2011). Differential effects of intrauterine growth restriction on brain structure and development in preterm infants: A magnetic resonance imaging study. Brain Res..

[21] Narberhaus A., Segarra D., Caldú X., Giménez M., Junqué C., Pueyo R., Botet F. (2007). Gestational age at preterm birth in relation to corpus callosum and general cognitive outcome in adolescents. J. Child Neurol..

[22] Giménez M., Junqué C., Narberhaus A., Botet F., Bargalló N., Mercader J.M. (2006). Correlations of thalamic reductions with verbal fluency impairment in those born prematurely. Neuroreport.

[23] Giménez M., Junqué C., Narberhaus A., Bargalló N., Botet F., Mercader J.M. (2006). White matter volume and concentration reductions in adolescents with history of very preterm birth: A voxel-based morphometry study. Neuroimage.

[24] Thompson D.K., Matthews L.G., Alexander B., Lee K.J., Kelly C.E., Adamson C.L., Hunt R.W., Cheong J.L.Y., Spencer-Smith M., Neil J.J. (2020). Tracking regional brain growth up to age 13 in children born term and very preterm. Nat. Commun..

[25] Peterson B.S., Vohr B., Staib L.H., Cannistraci C.J., Dolberg A., Schneider K.C., Katz K.H., Westerveld M., Sparrow S., Anderson A.W. (2000). Regional brain volume abnormalities and long-term cognitive outcome in preterm infants. J. Am. Med. Assoc..

[26] Ment L.R., Kesler S., Vohr B., Katz K.H., Baumgartner H., Schneider K.C., Delancy S., Silbereis J., Duncan C.C., Constable R.T. (2009). Longitudinal brain volume changes in preterm and term control subjects during late childhood and adolescence. Pediatrics.

[27] Parker J., Mitchell A., Kalpakidou A., Walshe M., Jung H.-Y., Nosarti C., Santosh P., Rifkin L., Wyatt J., Murray R.M. (2008). Cerebellar growth and behavioural & neuropsychological outcome in preterm adolescents. Brain.

[28] Karolis V.R., Froudist-Walsh S., Kroll J., Brittain P.J., Tseng C.E.J., Nam K.W., Reinders A.A.T.S., Murray R.M., Williams S.C.R., Thompson P.M. (2017). Volumetric grey matter alterations in adolescents and adults born very preterm suggest accelerated brain maturation. Neuroimage.

[29] de Kieviet J.F., Zoetebier L., van Elburg R.M., Vermeulen R.J., Oosterlaan J. (2012). Brain development of very preterm and very low-birthweight children in childhood and adolescence: A meta-analysis. Dev. Med. Child Neurol..

[30] Volpe J.J. (2019). Dysmaturation of premature brain: Importance, cellular mechanisms, and potential interventions. Pediatr. Neurol..

[31] Back S.A., Han B.H., Luo N.L., Chricton C.A., Xanthoudakis S., Tam J., Arvin K.L., Holtzman D.M. (2002). Selective vulnerability of late oligodendrocyte progenitors to hypoxia-ischemia. J. Neurosci..

[32] Segovia K.N., McClure M., Moravec M., Luo N.L., Wan Y., Gong X., Riddle A., Craig A., Struve J., Sherman L.S. (2008). Arrested oligodendrocyte lineage maturation in chronic perinatal white matter injury. Ann. Neurol..

[33] Nosarti C., Giouroukou E., Healy E., Rifkin L., Walshe M., Reichenberg A., Chitnis X., Williams S.C.R., Murray R.M. (2008). Grey and white matter distribution in very preterm adolescents mediates neurodevelopmental outcome. Brain.

[34] Morsing E., Malova M., Kahn A., Lätt J., Björkman-Burtscher I.M., Maršál K., Ley D. (2018). Brain volumes and developmental outcome in childhood following fetal growth restriction leading to very preterm birth. Front. Physiol..

[35] Manjón J.V., Coupé P. (2016). volBrain: An online MRI brain volumetry system. Front. Neuroinform..

